# Detection of Oxytetracycline in Citrus Phloem and Xylem Saps Using Europium-Based Method

**DOI:** 10.3390/antibiotics10091036

**Published:** 2021-08-25

**Authors:** Faraj Hijaz, Yasser Nehela, Ozgur Batuman, Nabil Killiny

**Affiliations:** 1 Department of Plant Pathology, Citrus Research and Education Center, Institute of Food and Agricultural Sciences, University of Florida, Lake Alfred, FL 33850, USA; fhijaz@ufl.edu (F.H.); yasser.nehela@ufl.edu (Y.N.); 2 Department of Agricultural Botany, Faculty of Agriculture, Tanta University, Tanta 31512, Egypt; 3 Department of Plant Pathology, Southwest Florida Research and Education Center, Institute of Food and Agricultural Sciences, University of Florida, Immokalee, FL 34142, USA; obatuman@ufl.edu

**Keywords:** oxytetracycline, huanglongbing, europium, phloem, xylem, antibiotic, citrus

## Abstract

Oxytetracycline (OTC) has been used for the control of several plant diseases and was recently approved for the control of Huanglongbing, the citrus greening disease. Huanglongbing is caused by the phloem limited ‘*Candidatus* Liberibacter asiaticus’. Determination of OTC in the xylem and phloem of citrus plants is of great interest as they are the main routes of translocation in citrus. In addition, the determination of the level of OTC in the phloem sap is necessary for the control of the ‘*Ca.* L. asiaticus’ pathogen, which resides in the phloem. Herein, we demonstrated that the level of OTC in the citrus phloem and xylem saps obtained using the centrifugation method can be successfully measured using the europium (Eu) method directly or with cleanup by solid-phase extraction (SPE). Recovery of OTC from spiked sap samples purified by solid-phase extraction (SPE) was higher than 90%, while recovery from saps without SPE cleanup were nearly 100%. The ‘*Ca.* L. asiaticus’-infected leaf and phloem sap samples showed higher inhibition of the fluorescence intensity of the OTC standard compared to non-infected control leaf and phloem samples. In agreement with this finding, the levels of phenols and flavonoids in ‘*Ca.* L. asiaticus’-infected leaves were higher than those controls and were shown to interfere with the Eu method. Therefore, the SPE cleanup step only improved OTC recovery from leaf samples containing the interfering compounds. The Eu method was then used to determine OTC levels in the phloem and xylem sap of OTC-treated plants, and the results were similar whether measured directly or after SPE. Visualization under ultraviolet light (400 nm) showed the presence of OTC in citrus xylem and phloem saps with and without the use of SPE.

## 1. Introduction

Huanglongbing, also known as citrus greening disease, is caused by a fastidious, phloem-limited, plant pathogenic bacterium ‘*Candidatus* Liberibacter’ spp. Three Liberibacter species are associated with HLB; ‘*Ca*. L. africanus’ in Africa, ‘*Ca*. L. americanus’ in Brazil, and ‘*Ca*. L. asiaticus’ in the Americas [1]. The ‘*Ca*. L. asiaticus’ is considered the most dangerous species as it is the most dominant, causing massive destruction to the citrus industry in different countries [1]. The ‘*Ca*. L. africanus’ is transmitted by the African psyllid citrus, *Trioza erytreae* Del Guercio (Hemiptera: Triozidae), whereas ‘*Ca*. L. americanus’ and ‘*Ca*. L. asiaticus’ are transmitted by the Asian citrus psyllid, *Diaphorina citri* Kuwayama (Hemiptera: Liviidae). The ‘*Ca.* L. asiaticus’-infected trees show a wide range of symptoms including blotchy and mottled leaves [1], lopsided, small-sized, asymmetric, bitter, and sour fruits. The roots of ‘*Ca.* L. asiaticus’-infected trees are poorly developed with very few fibrous roots, and infected trees look stunted and smaller than healthy trees. Infected trees may also develop twig and limb dieback, which often result in the tree’s death.

Oxytetracycline (OTC) and streptomycin have been efficiently used for the control of several plant diseases for more than 70 years [2]. For instance, OTC has been used for the control of several plant diseases including fire blight and spot disease on peaches, the yellow disease in elm and coconut palm trees, and the control of several bacterial pathogens on vegetables [3]. However, the development of streptomycin resistance restricted the use of antibiotics in the field [3].

Recently, streptomycin and OTC have been approved as a foliar spray for the control of Huanglongbing in citrus fields [4]. The approval was issued after the substantial losses in the citrus industry due to the complexity of the management of this disease. Management of Huanglongbing mainly depends on the control of *D. citri* using insecticides. Other management strategies, such as enhanced nutritional programs, thermotherapy, and removal of infected trees, were proposed for the control of Huanglongbing disease. However, these strategies were not useful in the field [5]. The use of antibiotics was recommended for the control of Huanglongbing disease in the early 1970s, and early studies showed trunk-injection of tetracycline could reduce the disease symptoms [5]. Recent studies also showed that streptomycin, oxytetracycline, penicillin, ampicillin, and several other antibiotics were effective against ‘*Ca.* L. asiaticus’ pathogen [6,7,8,9,10]. The previous studies indicated that antibiotics can be successfully used for the control of HLB.

Recently, we studied the translocation and persistence of OTC and streptomycin in citrus plants after root drench and stem application [5]. Our results showed OTC and streptomycin were translocated to the leaves, phloem (the outer bark), and xylem (the woody cylinder of the inner bark) after root-drench and stem application. The level of OTC detected in the canopy after root drench was lower than that of streptomycin, whereas the level of streptomycin in the root was higher than that of OTC after root drench. This result indicated that streptomycin could bind to xylem tissues [5]. The presence of OTC and streptomycin in the phloem, where the ‘*Ca.* L. asiaticus’ dwells, indicated that these antibiotics could be effective against this pathogen [5].

In another study, we investigated the movement of OTC in citrus plants upon root drench and trunk injection using girdled and non-girdled citrus plants [11]. Upon treatment, OTC was detected in the cortex (phloem), the inner part of the stem (xylem), and leaves above and below the girdled area [11]. The previous result indicated that the OTC was mainly translocated via the xylem. The occurrence of OTC beyond the girdling indicated that movement into the phloem occurs following the xylem translocation [11]. We also compared the efficiency of trunk injection and foliar application of OTC and investigated the effect of adjuvants on OTC uptake after foliar application [12]. Our results showed that trunk injection of OTC was better than foliar application [12]. Trunk injection resulted in high levels of OTC and significantly reduced the ‘*Ca.* L. asiaticus’ titer in treated plants. The addition of the adjuvants to the OTC solution did not improve its uptake [12].

Recently, we developed a fluorometric method for the detection of OTC based on its complexation with europium (Eu) and cetyltrimethylammonium chloride (CTAC), as a co-ligand [13]. Our results showed that the developed method can be used to estimate the level of OTC in citrus leaves. We also applied the new method in the field and compared it to the enzyme-linked immunosorbent assay (ELISA). The results obtained by the fluorometric method were similar to those found by the ELISA assay [13]. Similar results were also obtained after the replacement of CTAC with citrate (Cit) [14].

In this study, we employed the Eu method to measure the OTC levels in the phloem and xylem saps of healthy and ‘*Ca.* L. asiaticus’-infected plants. The level of OTC in phloem and xylem saps was determined without and with cleanup by SPE to remove matrix interferences. We showed that the SPE step was not critical for sap samples compared to leaf samples, which contain interfering compounds such as phenols and flavonoids. Therefore, OTC can be directly measured in citrus saps without any cleanup step, which means less costly, more accurate, and faster analysis.

## 2. Results

### 2.1. Recovery of OTC from Citrus Leaves Using the Acidic Extract and SPE

**Figure 1:**
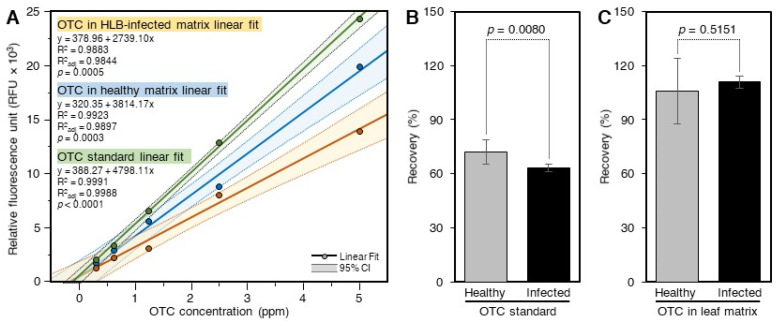
**Recovery of OTC from spiked citrus leaves.** (**A**) Standard curves of OTC in 60% methanol and citrus leaf extract of healthy and ‘*Ca.* L. asiaticus’-infected plants. The standard curves were prepared in the sample matrix by spiking the leaf extracts with OTC and then passing them through SPE. (**B**) Recovery of OTC from spiked ‘*Ca.* L. asiaticus’-infected and healthy leaves as calculated using the pure standard. (**C**) Recovery of OTC from spiked ‘*Ca.* L. asiaticus’-infected and healthy leaves as calculated using the standards prepared in the sample matrices and cleaned by SPE. Data are the means ± SD of five replicates (*n* = 5). Values with *p*-values < 0.05 are significantly different using a two-tailed student *t*-test.

The response of the standard curve generated using pure OTC in 60% methanol was higher than those prepared in the sample matrix and cleaned using SPE (Figure 1A). The standard curve generated in the matrix of control leaves was also higher than that generated in ‘*Ca.* L. asiaticus’-infected leaves (Figure 1A). The recovery of OTC from ‘*Ca.* L. asiaticus’-infected leaves (63.3 ± 1.9%), as estimated using the pure standard, was significantly lower than that obtained from control (72.1 ± 6.6%) leaves, indicating higher inhibition by ‘*Ca.* L. asiaticus’-infected leaves (Figure 1B). The recoveries of OTC from spiked control and ‘*Ca.* L. asiaticus’-infected leaves as determined using the OTC standard generated in the sample matrixes were higher than those calculated using the pure standard curve (Figure 1C). In addition, the recovery of OTC from control (105.7 ± 18.3%) leaves was similar to that from ‘*Ca.* L. asiaticus’-infected (110.9 ± 3.3%) when calculated using the standard curves generated in the plant matrix of control and ‘*Ca.* L. asiaticus’-infected plants (Figure 1C).

### 2.2. Level of Phenols and Flavonoids in Control and ‘Ca. L. asiaticus’-Infected Leaves

**Figure 2:**
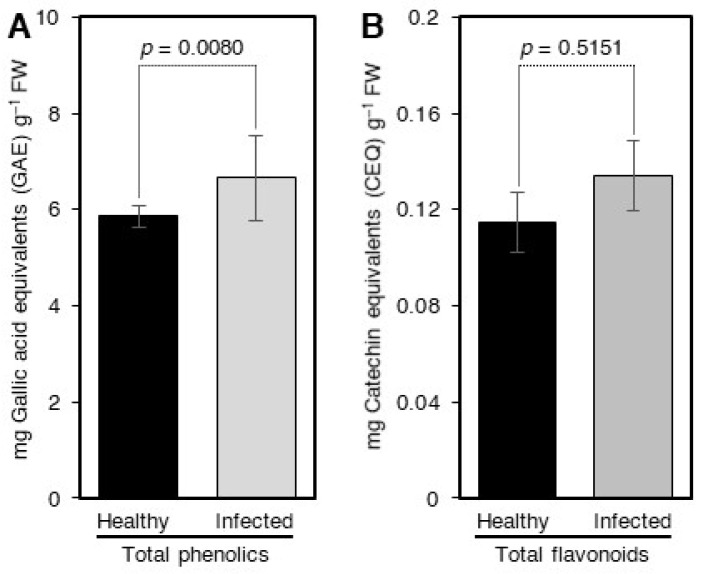
**The level total phenols (A) and flavonoids (B) in ‘*Ca.* L. asiaticus’—infected and healthy leaves.** Data are the means ± SD of five replicates (*n* = 5). Values with *p*-values < 0.05 are significantly different using a two-tailed student *t*-test.

The high inhibition of the fluorescence intensity of the OTC standard complex in the ‘*Ca.* L. asiaticus’-infected leaves indicated that the levels of phenols and flavonoids in ‘*Ca.* L. asiaticus’-infected plants were higher than those of control plants. In agreement with the previous assumption, our results showed that the levels of phenols and flavonoids in ‘*Ca.* L. asiaticus’-infected leaves were significantly higher than those of control leaves (Figure 2A,B).

### 2.3. Recovery of OTC from Citrus Phloem Sap Using the Acidic Extract and SPE

**Figure 3:**
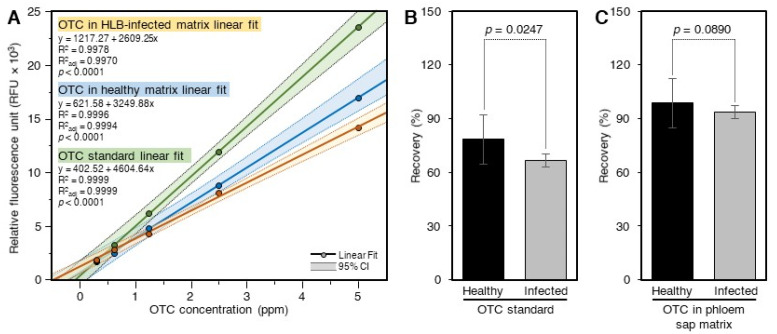
**Recovery of OTC from spiked citrus phloem sap.** (**A**) Standard curves of OTC in 60% methanol and citrus phloem sap obtained from healthy and ‘*Ca.* L. asiaticus’-infected plants. The standard curves were prepared in the phloem sap matrix by spiking phloem sap extracts with OTC and then passing through the SPE cartridge. (**B**) Recovery of OTC from spiked ‘*Ca.* L. asiaticus’-infected and healthy phloem sap as calculated using the pure standard. (**C**) Recovery of OTC from spiked ‘*Ca.* L. asiaticus’-infected and healthy phloem sap as calculated using the standards prepared in the phloem sap extracts of healthy and ‘*Ca.* L. asiaticus’-infected plants and cleaned using SPE. Data are the means ± SD of five replicates (*n* = 5). Values with *p*-values < 0.05 are significantly different using a two-tailed student *t*-test.

The response of the standard curve generated using OTC in 60% methanol was higher than those prepared in the phloem sap matrix and cleaned using SPE (Figure 3A). The response of the standard curve generated in the phloem sap of control plants was higher than that generated in the phloem sap of ‘*Ca.* L. asiaticus’-infected plants (Figure 3A). The recovery of OTC from the phloem sap of ‘*Ca.* L. asiaticus’-infected plants (66.6 ± 3.7%) as estimated using the pure standard was significantly lower than that obtained from non-infected control (78.4 ± 13.7%) plants (Figure 3B). The recoveries of OTC from spiked control (97.7 ± 5.9%) and ‘*Ca.* L. asiaticus’-infected (93.0 ± 6.0%) phloem saps, as determined using the OTC standard generated in the phloem saps and cleaned using SPE, were similar to each other and higher than those calculated using the pure standard curve (Figure 3C).

### 2.4. Recovery of OTC from Citrus Xylem Sap Using the Acidic Extract and SPE

**Figure 4:**
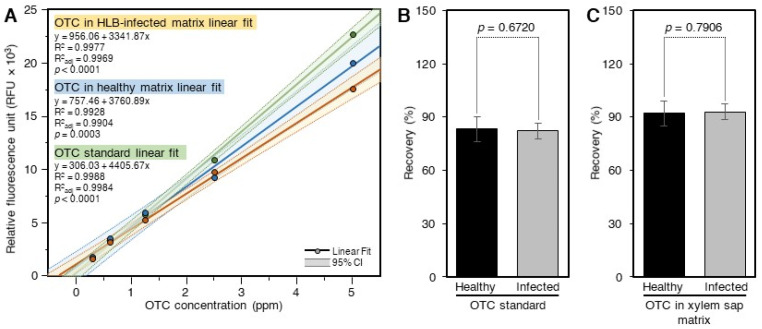
**Recovery of OTC from spiked citrus xylem sap.** (**A**) Standard curves of OTC in 60% methanol and citrus xylem sap obtained from healthy and ‘*Ca.* L. asiaticus’-infected plants. The standard curves in the phloem sap matrix were prepared by spiking xylem sap extracts with OTC and then purifying them using SPE. (**B**) Recovery of OTC from spiked ‘*Ca.* L. asiaticus’-infected and healthy xylem sap as calculated using the pure OTC standard curve. (**C**) Recovery of OTC from spiked ‘*Ca.* L. asiaticus’-infected and healthy xylem sap as calculated using the OTC standards prepared in the xylem sap extracts and cleaned using SPE. Data are the means ± SD of five replicates (*n* = 5). Values with *p*-values < 0.05 are significantly different using a two-tailed student *t*-test.

The response of the standard curve generated using 60% methanol was higher than those prepared in the xylem saps and cleaned using SPE (Figure 4A). The response of the standard curve generated in the xylem sap of control plants was slightly higher than that generated in the xylem sap of ‘*Ca.* L. asiaticus’-infected plants (Figure 4A). The recovery of OTC from the xylem sap of ‘*Ca.* L. asiaticus’-infected plants (82.1 ± 4.3%), as estimated using pure standard, was similar to that obtained from the control (83.3 ± 7.0%) plants (Figure 4B), indicating that the xylem composition of ‘*Ca.* L. asiaticus’-infected plants was not affected by the ‘*Ca.* L. asiaticus’ infection. The recoveries of OTC from spiked control (92.1 ± 8.2%) and ‘*Ca.* L. asiaticus’-infected (93.0 ± 5.3%) xylem sap, as determined using the OTC standard generated in the xylem saps and cleaned using the SPE, were similar to each other and higher than those calculated using the pure standard curve (Figure 4B).

### 2.5. Direct Recovery of OTC from Citrus Phloem and Xylem Saps

**Figure 5:**
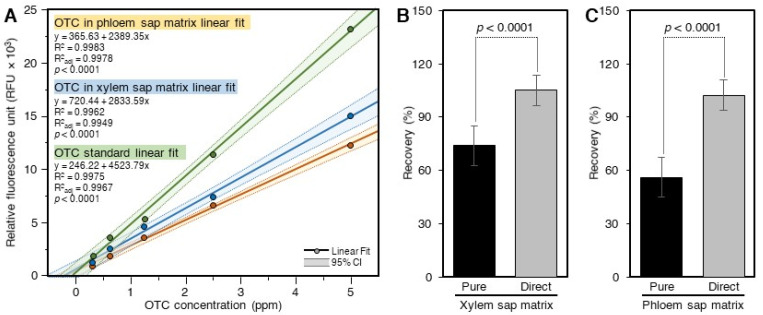
**Recovery of OTC from spiked citrus xylem and phloem saps by direct analysis (without SPE).** (**A**) Standard curves of OTC in 60% methanol and citrus xylem and phloem sap. The standard curves in the sap matrices were prepared by spiking the xylem and the phloem sap with OTC and analyzing it directly after being diluted with 60% methanol. (**B**) Recovery of OTC from spiked ‘*Ca.* L. asiaticus’-infected and healthy xylem sap as calculated using the pure OTC standard and the standards prepared in the xylem sap matrix and measured directly without the SPE. (**C**) Recovery of OTC from spiked phloem sap as calculated using the pure OTC standard and the standards prepared in the phloem sap matrix and measured without the SPE. Data are the means ± SD of five replicates (*n* = 5). Values with *p*-values < 0.05 are significantly different using a two-tailed student *t*-test.

The OTC standard curves prepared in the xylem and phloem saps showed about 34 and 46% inhibition, respectively, compared to the pure standard (Figure 5A). The recovery of OTC from the xylem (73.9 ± 11.1) sap, as estimated using the pure standard, was lower than that analyzed using the standard curve prepared in the xylem sap matrixes 105.2 ± 8.6% (Figure 5B). Likewise, the recovery of OTC from the phloem sap (56.1 ± 2.1%) as estimated using the pure standard was lower than those calculated using the standard curve prepared in the phloem sap matrices (102.3 ± 3.2%) (Figure 5C).

### 2.6. Analysis of OTC in the Phloem and Xylem Sap of Treated Plants

**Figure 6:**
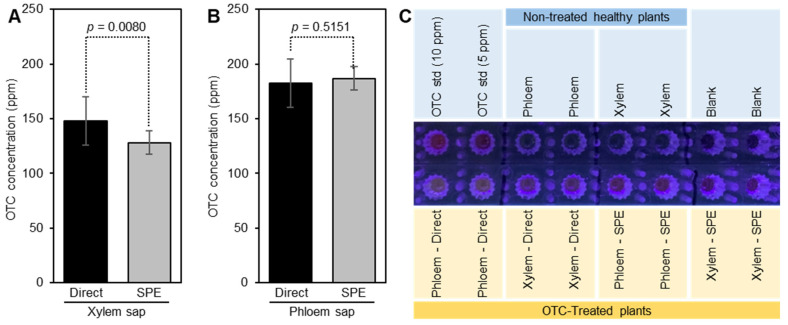
**Determination and visualization of OTC in the phloem and xylem saps of OTC-treated plants.** (**A**) The level of OTC in the xylem sap as measured using the direct method (without SPE) and after SPE. (**B**) The level of OTC in the phloem sap as measured using the direct method (without SPE) and after SPE. Data are the means ± SD of five replicates (*n* = 5). Values with *p*-values < 0.05 are significantly different using a two-tailed student *t*-test. (**C**) Fluorescence imaging of OTC in standards and phloem and xylem saps of OTC-treated plants. Saps were obtained by centrifugation and extracted using 1 M HCl containing 2.2% trichloroacetic acid, and either cleaned using SPE or measured directly. Samples were placed in a 96-well microplate and their image was taken under ultraviolet light (400 nm).

To verify that we can measure OTC in the phloem and xylem saps of treated citrus, we incubated several stems in an OTC solution, and then extracted the phloem and xylem saps. High levels of OTC were detected in the xylem and phloem sap of treated plants (Figure 6A,B). The level of OTC in the xylem sap was 147.8 ± 21.9 ppm and 128.5 ± 12.5 as determined directly or after the use of SPE, respectively (Figure 6A). The level of OTC in the phloem sap was 182.8 ± 21.9 and 186.9 ± 6.1 ppm as determined directly or after the use of SPE, respectively (Figure 6B). These results indicated that the level of OTC in citrus sap can be measured directly without a cleanup step, however, the standard should be prepared in the plant sap to compensate for the matrix effect.

### 2.7. Visualization of OTC in the Phloem and Xylem Sap of Treated Plants

Our results showed that the OTC in the phloem sap and xylem sap of citrus plants can be directly visualized under ultraviolet light (400 nm) by complexing it with Eu(III) and citrate (Figure 6C). Although direct visualization of OTC in the phloem sap was possible, the fluorescence intensity in phloem sap after the SPE was higher than without the SPE (Figure 6C).

## 3. Discussion

The xylem and the phloem are the main routes for the translocation of agrochemicals. Translocation in the xylem occurs upward in the direction of the transpiration stream, whereas phloem movement can be upward (acropetal) or downward (basipetal) following the source to sink direction [15]. Translocation of agrochemicals can occur via the xylem or the phloem or by both routes (ambimobile) [15]. Xylem movement is the most efficient way for the translocation of insecticides as it results in a consistent distribution throughout the canopy, whereas the phloem movement is best for herbicides as it results in the accumulation of herbicides in the new shoots [15]. The efficiency of agrochemicals depends on their capability to reach their targets.

Similarly, the efficiency of applied antibiotics in planta also depends on their capability to reach their target. For example, several antibiotics were effective against spiroplasmas and phytoplasmas in vitro. However, only oxytetracycline was effective against these pathogens in planta, indicating that it can reach the phloem [16]. Using girdled citrus seedlings and trees, we showed that the xylem was the main route for OTC transportation in citrus plants [11]. The presence of OTC in phloem tissues beyond the girdled area indicated a bidirectional movement between the xylem and the phloem [11]. In addition, the determination of the level of OTC in the xylem and the phloem sap is critical for the control of ‘*Ca.* L. asiaticus’, which resides in the phloem of its host plants.

In our previous studies, we established a fluorometric method for the measurement of OTC in citrus leaves by complexing it with Eu using CTAC or citrate as sensitizing agents [13,14]. Our previous studies showed that the citrus matrix significantly reduced the fluorescence intensity of the Eu-OTC complex. Our previous investigation showed that phenols and flavonoids were behind this inhibition [13,14]. Our current results confirmed our previous findings and showed that the fluorescence intensity of the Eu-OTC-Cit in the matrix of the ‘*Ca.* L. asiaticus’-infected leaves was lower than that in control leaves. In agreement with this result, the colorimetric assays showed that the levels of phenols and flavonoids in ‘*Ca.* L. asiaticus’-infected leaves were significantly higher than those of the control leaves.

Our current results demonstrated that the europium-based method could also be used to measure OTC levels in the phloem and xylem saps obtained from citrus stems by centrifugation. Low recovery of OTC from phloem sap of ‘*Ca.* L. asiaticus’-infected (67%) and control (78%) plants was obtained using the standard curve of OTC in methanol. On the other hand, higher recoveries of OTC (93–98%) were obtained using the standard curves that were prepared in the phloem sap matrix of control and ‘*Ca.* L. asiaticus’-infected plants. These results indicated that the standard curve should be prepared in the sample matrix to compensate for the matrix inhibition. Similar recoveries of OTC were obtained from the xylem citrus sap. The recoveries of OTC from ‘*Ca.* L. asiaticus’-infected and control xylem as calculated using the pure standard were good (~83%) and increased to ~93% when using the OTC standards prepared in the xylem matrix.

Our results showed that the level of OTC in the citrus phloem and xylem sap can be measured directly without SPE. The standard curve prepared in the xylem sap showed less inhibition (~34%) compared to that prepared in the phloem sap (~46%). In agreement with this result, low OTC recoveries were also obtained from spiked phloem (~56%) and xylem (~74%) sap using the pure OTC standard curve. On the other hand, these recoveries reached ~100% when the recoveries were calculated using the standards prepared in the sap matrices. This result showed that OTC can be directly measured in the citrus phloem and xylem sap if the standard curves were prepared in the same matrix as the samples.

To show that we can measure the level of OTC in the phloem and xylem sap of citrus plants, we incubated several citrus stems in OTC solution, and we analyzed them with and without the SPE step. Our results showed that the level of OTC in the phloem and xylem sap of OTC-treated plants can be successfully determined with or without a cleanup step. The levels of OTC in the phloem and xylem saps measured directly without solid-phase extraction were similar to those measured after the SPE. Our results also showed that visualization of OTC in the phloem and xylem sap can be achieved using UV light (400 nm) with or without SPE. However, the intensity of the pink color of the OTC-Eu-Cit complex was higher after using the SPE.

## 4. Material and Method

### 4.1. Extraction of OTC from Spiked Leaves

Leaves were collected from two-year-old Valencia sweet orange (*Citrus sinensis* (L.) Osbeck). The ‘*Ca*. L. asiaticus’ infection was confirmed using PCR analysis [12]. Citrus leaves were ground in liquid nitrogen [13], and 100-mg of the ground tissues was spiked with 50 µL of OTC standard (200 ppm) [13]. Five leaf samples from each treatment, non-infected control and ‘*Ca*. L. asiaticus’-infected leaves, were spiked with OTC standard. The OTC was extracted using 1 M HCl including 2.2% trichloroacetic acid as reported previously [13]. The sample extracts were cleaned up using an Oasis hydrophilic-lipophilic balance (HLB) cartridge (3 cc, 60 mg, Waters, Milford, MA, USA) as described in our previous study [13]. Three standard curves were prepared; one was prepared in 60% methanol and measured directly without solid-phase extraction, and the other two were prepared in the sample matrix (acidic extracts of control and ‘*Ca*. L. asiaticus’-infected leaves) and cleaned using solid-phase extraction (SPE).

### 4.2. Extraction of Phenolics and Flavonoids from Citrus Leaves

Phenols and flavonoids were extracted from ‘*Ca*. L. asiaticus’-infected and control leaves using methanol as described in our previous work [17]. The levels of total phenols were measured using Folin–Ciocalteu reagents and the levels of total flavonoids were measured using aluminum chloride assay as described earlier [17]. Total phenols were reported as mg gallic acid equivalents (GAE) g^−1^ fresh weight (FW) and total flavonoids as mg catechin equivalents (CEQ) g^−1^ FW sample [17].

### 4.3. Collection of Citrus Phloem and Xylem Sap

**Figure 7:**
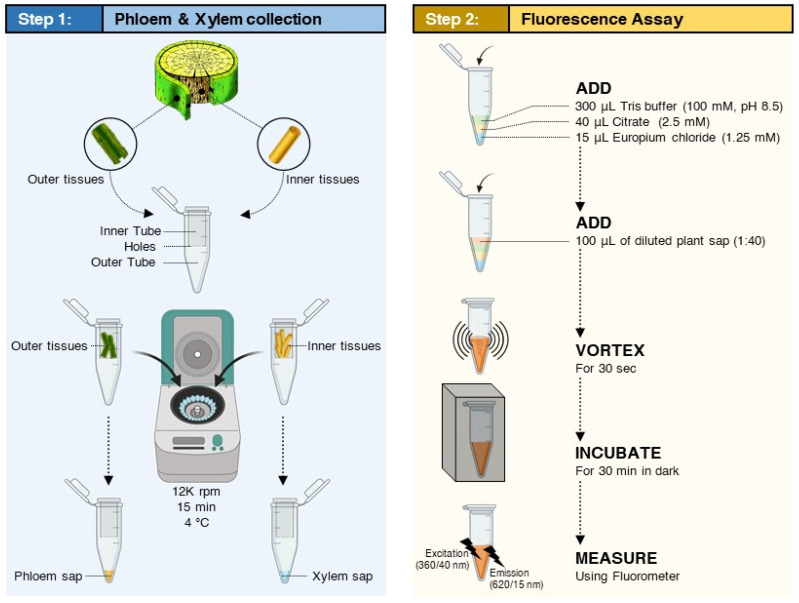
**Flow chart of the determination of OTC in citrus sap phloem and xylem sap (directly without using SPE). Step 1**: Collection of citrus phloem and xylem saps by centrifugation. **Step 2**: Determination of OTC using the fluorescence assay by mixing diluted saps (1 to 40) with Eu and citrate reagents in Tris buffer (100 mM, pH 8.5).

Stems (10–20 cm, ~0.5 cm diameter) were collected from healthy and ‘*Ca.* L. asiaticus’-infected Valencia sweet orange trees (2 years old). The phloem and xylem saps were collected by the centrifugation method (Figure 7) developed in our previous studies [18,19]. Briefly, the bark was manually removed from the twig using a sharp blade. The outer stem (phloem) tissues and the inner stem (xylem) tissues were rinsed with distilled water and dried with Kimwipes™. The bark was cut into small pieces (~1 cm long) and were vertically packed into a 0.5-mL Eppendorf tube with the bottom of the tube punctured to allow the sap to pass through. The 0.5-mL tube was placed into a 2-mL tube and was centrifuged for 15 min at 12,000 rpm at 4 °C (Figure 7). The collected phloem sap was stored at −80 °C until analysis. The xylem sap was collected from the inner tissues in the same way. Five plants were sampled from each treatment and about 200 µL was collected from each plant.

### 4.4. Extraction of OTC from Spiked Saps

A 100-µL aliquot of the phloem or xylem sap was spiked with 50 µL of OTC standard (200 ppm). Controls were spiked with 50 µL of distilled water. Five samples from each treatment were spiked with the OTC standard. The OTC was extracted using the acidic solution as described in our previous study [13]. The OTC in the sample extract was cleaned using SPE as described in the previous section. Three standard curves were prepared for each sap; one was prepared in 60% methanol and measured directly without SPE, and the other two were prepared in the sap matrices (extracts of control and ‘*Ca.* L. asiaticus’-infected saps) and cleaned using SPE.

### 4.5. Direct Analysis of OTC in Citrus Sap

The standard curve was prepared in the citrus sap and analyzed directly without SPE. Briefly, 50 µL of the citrus sap was mixed with 50, 25, 12.5, or 6.2 µL of 200 ppm OTC standard, and the final volume was completed to 2 mL using 60% methanol. The pure OTC standard was prepared in 60% methanol. To determine the percent (%) recovery, the phloem or the xylem sap was spiked with 25 µL of 200 ppm OTC standard and analyzed in the same way.

### 4.6. Determination of OTC Levels in the Xylem and Phloem Sap of Treated Plants

Ten Valencia stems (15–25 cm) were incubated in 200 ppm OTC solution for 96 h. At the end of the incubation time, the stems were washed three times with distilled water and dried using Kimwipes™. The phloem and xylem saps were collected using centrifugation as described above. OTC was extracted from the xylem and phloem sap using the acidic solution and cleaned using the SPE as described above. In addition, the level of OTC in the collected saps was analyzed directly (without SPE cleanup) after being diluted (10 µL to 400 µL) in 60% methanol.

### 4.7. Fluorescence Assay

The assay was performed using 300 µL tris buffer (100 mM, pH 8.5), 100 µL standard or sample, 40 µL of 2.5 mM citrate, and 15 µL of europium chloride (1.25 mM) (Figure 7). The fluorescence intensity was read after 30 min using a Synergy Multimode reader fluorometer (Bioteck, Winooski, VT, USA). The gain was set to 80 and the excitation and emission wavelengths were set to 360 ± 40 nm and 620 ± 20 nm (Figure 7), respectively [13].

### 4.8. Statistical Analysis

JMP Pro 15.0 software (SAS, Cary, NC, USA) was used for the statistical analysis. The % recoveries of OTC from control and ‘*Ca.* L. asiaticus’-infected plants were compared to each other using a two-tailed *t*-test (*p* < 0.05). The % recovery of OTC from plant saps calculated using the pure standard was compared to that determined using the standard prepared in plant matrix and measured directly using a two-tailed *t*-test (*p* < 0.05). The level of OTC in the phloem and xylem saps in OTC-incubated plants calculated using the pure standard were compared to those obtained using the standard prepared in plant matrix and cleaned with SPE using a two-tailed *t*-test (*p* < 0.05).

## 5. Conclusions

In the current study, we showed that the level of OTC in the phloem and xylem saps can be determined using the europium method, with or without using the SPE cleanup cartridge. SPE cleanup is not required for citrus saps due to a lack of the matrix-interfering found previously for leaves using this method. In addition, we were able to visualize the OTC in the phloem and xylem saps under ultraviolet light in samples both with and without SPE cleanup. The levels of OTC in the xylem and phloem of citrus plants confirm that they are the main routes for OTC movement. In addition, the determination of OTC levels in the phloem is of great interest, as ‘*Ca.* L. asiaticus’ is a phloem-restricted pathogen.

## Data Availability

Data are contained within the article.
